# Advanced radiomic prediction of osteoporosis in primary hyperparathyroidism: a machine learning-based analysis of CT images

**DOI:** 10.1007/s11547-025-02004-z

**Published:** 2025-04-24

**Authors:** Antonio Adarve-Castro, Virginia Soria-Utrilla, José Miguel Castro-García, María Dolores Domínguez-Pinos, Francisco Sendra-Portero, Miguel J. Ruiz-Gómez, José Algarra-García

**Affiliations:** 1 Departamento de Radiología y Medicina Física, Facultad de Medicina, Universidad de Málaga, Bulevar Louis Pasteur, 32, 29010 Málaga, Spain; 2 University Hospital Virgen de la Victoria, Málaga, Spain; 3 Regional University Hospital of Malaga, Málaga, Spain; 4 General University Hospital of Alicante, Alicante, Spain

**Keywords:** Osteoporosis, Bone mineral density, Hyperparathyroidism, Radiomic textures, Machine learning algorithms

## Abstract

This study aims to assess the proficiency of supervised machine learning techniques in discriminating between normal and abnormal bone mineral density (BMD) by leveraging clinical features and texture analysis of spinal bone tissue in patients diagnosed with primary hyperparathyroidism (PHP). From a total of 219 patients diagnosed with PHP, the 58 who had undergone both DXA and abdominal CT scan were included in this study. BMD was assessed by quantifying the Hounsfield units (HU) and performing texture analysis on every CT scan. The first lumbar vertebral body texture features were extracted by using LifeX 7.3.0 software. Initial classification into normal or abnormal BMD was performed with different machine learning techniques by training a model with the variables obtained from the texture analysis. Differentiating osteopenia from osteoporosis was evaluated by creating two models, one including the variables obtained from the texture analysis and HU and another one which only included the HU. Their performance was evaluated in the validation and test groups by calculating the accuracy, precision, recall, F1 score, and AUC. Bayes demonstrated higher performance for discerning individuals with normal and abnormal BMD, with an AUC of 0.916. The results from the second analysis showed a better performance for the model including the variables obtained from the texture analysis compared to the model that was solely trained with the HU (AUC in the training group of 0.77 vs. 0.65 in the test groups, respectively). In conclusion, analysis of BMD obtained from abdominal CT scans including texture analysis provide a better classification of normal density, osteopenia and osteoporosis in patients with PHP.

## Introduction and objectives

Primary hyperparathyroidism (PHP) is an endocrine disease caused by excessive secretion of parathyroid hormone (PTH) from the parathyroid glands, caused by a solitary parathyroid adenoma in 80–85% of cases. PHP affects 1% of the adult population, although its prevalence increases up to 2% in those over 55 years of age [1, 2].

PHP is associated with osteoporosis [3], which is defined as a “systemic skeletal disease characterized by low bone mass and microarchitectural deterioration of bone tissue with a consequent increase in bone fragility and susceptibility to fracture” [4]. For this reason, osteoporosis serves as a critical indicator for the surgical removal of the adenoma [2].

In Spain, there are approximately 285,000 new fragility fractures annually, equivalent to 782 fractures per day [5]. Overall, PHP represents approximately 3% of these pathological fractures [1]. However, despite fractures cause limitations in quality of life and increase morbidity and risk of mortality, osteoporosis is still underdiagnosed [4].

Currently, the measurement of bone mineral density (BMD) by dual-energy X-ray absorptiometry (DXA) is the gold standard for the diagnosis of osteoporosis [6]. In patients with PHP, prolonged exposure to elevated PTH levels results in a decrease in cortical BMD while trabecular BMD remains relatively preserved. This pattern suggests that patients with PHP might be at increased risk for distal radial fractures and reduced risk for vertebral fractures. However, numerous studies have demonstrated an overall increased fracture risk in PHP [1, 7].

In recent years, various authors have proposed alternative techniques for evaluating BMD beyond DXA. Several DXA-based software have been assessed in PHP. Trabecular bone score (TBS) is a novel numerical index that estimates trabecular bone microarchitecture using texture analysis of lumbar spine DXA images. The Sociedad Española de Investigación Ósea y del Metabolismo Mineral (SEIOMM) has recognized the clinical utility of TBS in PHP [7]. Bone strain index (BSI) is a new metric for bone strength based on finite element analysis from lumbar spine and femoral neck DXA images. BSI is impaired in PHP and may help to identify patients with PHP at high risk of fractures [8]. Other previous studies have explored the use of thoracic and abdominal CT scans, performed for other medical reasons, to assess spinal BMD through complex texture analysis of pixel data [9–12]. This approach not only has the potential to reduce the expense and time consumption of repetitive radiological tests but also minimizes patient displacement and radiation exposure [13]. However, there are no available studies in patients with PHP that have assessed spinal BMD through texture analysis.

Taken all this together, in this study we aimed to assess the performance of various supervised machine learning techniques in classifying normal versus abnormal BMD in patients with PHP, with a focus on mitigating additional expenses.

## Methods

### Study design

This retrospective study included all consecutive patients diagnosed with primary hyperparathyroidism (PHP) referred to the Radiology Department of Virgen de la Victoria Hospital in Malaga between June 2019 and January 2023. Eligible patients had undergone both dual-energy X-ray absorptiometry (DXA) and a CT scan for any reason, covering the lumbar spine region. We included CT scans performed within the 6 months prior to DXA to balance the need for a sufficient sample size with the requirement for temporal proximity between CT and DXA scans, as in previous studies [14]. Likewise, we included CT scans performed within one month after DXA to minimize the potential confounding effects of any post-DXA pharmacological interventions. Exclusion criteria included patients with significant movement artifacts potentially limiting radiological assessment.

The study was conducted following the guidelines of the Standards of Good Clinical Practice (art. 34 RD 223/2004; community directive 2001/20/EC) and the Declaration of Helsinki of the World Medical Association (Brazil, Fortaleza 2013) and Regulation (EU) 2016/679 of the European Parliament and of the Council of 27 April 2016 on the protection of individuals with regard to the processing of personal data and on the free movement of such data (Organic Law 3/2018 in Spain). Due to the retrospective nature of the study and the anonymization of patients, informed consent was not required. The study was approved by the Research Ethics Committee provincial of Málaga (reference number SICEIA-2024–001104).

### Image acquisition

CT studies utilized General Electric Revolution Evo equipment (64 and 128 detectors) with KV protocols from 100 to 120 and variable MAs from 100 to 400. Intravenous contrast, when administered, was calculated based on patient weight, with a concentration of 300 mg/ml. DXA studies were conducted at the lumbar spine and femoral neck using General Electric Healthcare™ equipment. CT images and DXA results were retrieved from the picture archiving and communication system (PACS).

### Density measurement and texture analysis

BMD values obtained by DXA are expressed as standard deviations (SD) from the normal range. The T-score was used for postmenopausal women or women aged over 50 and for men aged over 65, as it compares the patient's BMD with the mean BMD of young healthy individuals. The Z-score was used for premenopausal women under 50 and for men under 65, as it compares the patient's BMD with healthy controls of the same age. BMD values lower than -1 SD indicate osteopenia, while values below -2.5 SD confirm osteoporosis [3].

Bone density assessment involved semi-automatic segmentation with a region of interest (ROI) on the medial aspect of the L1 vertebral body or L2 in case of lesions use CT of lumbar spine in sagittal projection, excluding the cortical portion of the vertebra in all cases. A qualified radiologist with 5 years of experience in musculoskeletal radiology performed the analysis of all studies.

Texture analysis utilized DICOM-formatted images subjected to a normalization process (Laplacian of Gaussian filter) using ImageJTM software. This normalization technique has been utilized in previous studies to harmonize the distribution of pixels in CT scans with and without contrast, employing different KV and MA values during acquisition [15].

**Fig. 1:**
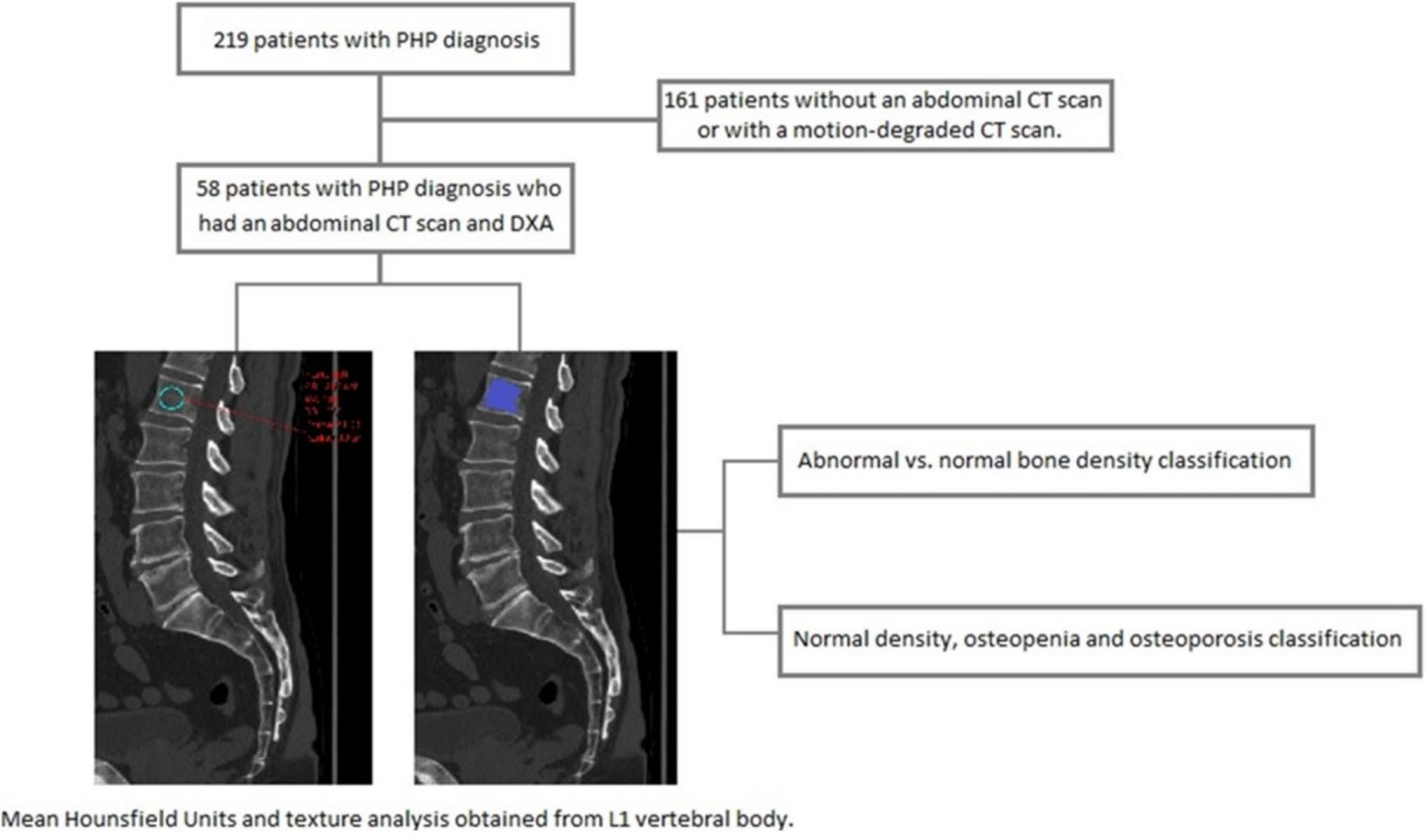
Study flowchart

Lifex 7.3.0TM software was employed for segmentation, followed by analysis of 38 first-order and 18 s-order statistics (Fig. 1). The extracted radiomic characteristics from each image were analyzed using SPSS 19.0™ (Statistical Package for the Social Sciences).

### Statistical analysis and model design

Hounsfield units (HU) and texture analysis-derived variables were compared between patients with normal and decreased (< −1 SD) densitometric values using the Mann–Whitney U test. Neural network, logistic regression, and Naive Bayes techniques were applied after random division into training (72.5%) and testing (27.5%) cohorts (Fig. 1). A tenfold cross-validation in the training group aimed to mitigate overfitting.

Neural network hyperparameters included 900 nodes, 2500 iterations, ReLu activation, and Adam solver. Logistic regression datasets were normalized using the Lasso technique with a maximum of 2500 iterations. An ensemble method combined the neural network technique with Naive Bayes through a hard voting classifier. Evaluation metrics (accuracy, precision, recall, F1 score, and AUC) were calculated for the test group. All analyses were implemented using Python version 3.8.8, scikit-learn version 0.24.2, seaborn version 0.12.2, and SHAP version 0.40.0.

A second analysis was performed in order to classify the patients with normal density, osteopenia or osteoporosis. A first model based only on the HU and a second one including HU and the variables derived from texture analysis were created. The variables included in the second model were: minimum gray level IBSI1GSF, intensity range IBSI 20JQ and small zone emphasis IBSI5QRC. The models were trained with 80% of the sample with a fivefold cross-validation and the performance was tested in the remaining 20% of the sample. Accuracy, precision, recall, F1, and AUC were evaluated for both the validation and the test group.

## Results

A total of 219 patients diagnosed with primary hyperparathyroidism (PHP) were included in the study. Among them, 58 patients underwent a CT scan covering the lumbar spine region within the specified timeframe around the dual-energy X-ray absorptiometry (DXA) test. Of these patients, 11 (18.9%) were male with a median age of 53 (49.5–58.2) years, and 47 (81.1%) were female with a median age of 57 (52.3–64.8) years.

In terms of DXA results, 24 (41.4%) patients had normal findings, while 34 (58.6%) had abnormal results. No statistically significant differences in age and sex were observed between these two groups (Table 1).

**Table 1:** Variables in patients with normal and abnormal DXA

Variables		Patients with normal DXA (*n* = 24)	Patients with abnormal DXA (*n* = 34)	*p*
Age (years)		55 (51–60.5)	56 (51.75–64.25)	> 0.05
Sex	Men	7 (29%)	4 (12%)	
	Women	17 (71%)	30 (88%)	> 0.05
Type of CT	without iv contrast	10 (42%)	14 (41%)	
	with iv contrast	14 (58%)	20 (59%)	> 0.05
HU values		153 ± 41.72	107 ± 33.04	< 0.001
	without iv contrast	149.63 ± 54.72	103.57 ± 26.92	0.015
	with iv contrast	156.67 ± 32.78	122.64 ± 50.63	0.041
	Difference	p > 0.05	p > 0.05	
Texture analysis	Median intensity	19 (18–23.75)	17 (15–19)	< 0.001
	Intensity-based 10th percentile	5 (4–7.75)	3.5 (2.75–5.25)	< 0.05
	Intensity-based 25th percentile	12 (10–14.75)	9.5 (8–12)	< 0.01
	Intensity-based 75th percentile	28 (25.25–32)	24.5 (23–27)	< 0.001
	Intensity-based 90th percentile	35 (32–40)	32 (29–34.25)	< 0.001
	Intensity-based _KurtosisIBSI:IPH6	0.6 (0.46–0,82)	0.53 (0.37–0.75)	< 0.01
	Intensity-based maximum gray level IBSI:84IY	93 (85–113.75)	81.5 (72–92)	< 0.01
	Intensity-based interquantile range IBSI:SALO	15 (15–18.5)	14 (14–16.26)	< 0.05
	Intensity-based Range IBSI:20JQ	93 (85–113.75)	81.5 (72–92)	< 0.01
	Intensity-based mean absolute deviation IBSI:4FUA	9.0051E15 (8.548E15-1.1146E16)	8.12135E + 15 (7.26E + 15–8.742E + 15)	< 0.01
	Intensity-based mean absolute deviation IBSI:N72L	8.9618E15 (8.3721E15-9.9117E15)	8.138E15 (5.4973E15-9.5189E15)	< 0.05
	Root mean squarel intensity IBSI: 5ZWQ	2.178E16 (7.4589E15-2.4135E16)	1.8066E16 (2.096E15-2.1828E16)	< 0.01
	GLRLM Gray level non-uniformity R5YN	222.75 (192.75–252)	235.88 (214.63–248)	< 0.01

Regarding the CT studies, 34 (58.6%) were performed with intravenous contrast, and 24 (41.4%) were conducted without it. The mean Hounsfield unit (HU) values for studies with and without contrast were 134.5 ± 36.5 and 124.5 ± 33.4, respectively, with no statistically significant differences between the two groups (*p* > 0.05).

In the evaluation of HU between normal and abnormal DXA groups, a cutoff point of 106 HU demonstrated a specificity of 90% and sensitivity of 61%, with an AUC of 0.84 (*p* < 0.001). Similarly, a cutoff point of 160 HU provided a specificity of 89.9% and sensitivity of 60%, with an AUC of 0.81 (*p* < 0.001). Significant differences in HU values were found between the normal and abnormal DXA groups (*p* < 0.001), with mean HU being 153 ± 41.72 in normal DXA and 107 ± 33.04 in abnormal DXA (Table 1).

### Texture analysis

A comprehensive set of 38 first-order variables and 18 s-order variables were obtained for all CT studies. Statistically significant differences were observed in various first-order and second-order variables when comparing patients with normal and abnormal DXA. (Table 1).

When analyzing the results of texture analysis separately for CT studies with and without contrast, no statistically significant differences were found between patients with normal and abnormal DXA (*p* > 0.05).

### Machine learning model performance

We developed a first model to discern between normal or abnormal BMD in the entire sample. To this end, the variables HU, Kurtosis (IPH6), maximum gray level (84IY), and gray level non-uniformity (GLRLM R5YN) were selected to train and test the four machine learning techniques.

Kurtosis (IPH6) measures the sharpness or peakedness of intensity distribution, reflecting variability in pixel intensities and highlighting differences within bone structure. Maximum gray level (84IY) represents the highest pixel intensity in the region of interest, identifying areas of maximum attenuation that may correspond to denser bone regions. Gray level non-uniformity (GLRLM R5YN), a second-order feature, captures the variability of gray levels across continuous pixel paths, emphasizing texture heterogeneity. These variables were selected based on their statistical significance (*p* < 0.01) in distinguishing normal from abnormal BMD and on their contribution to model performance.

**Fig. 2:**
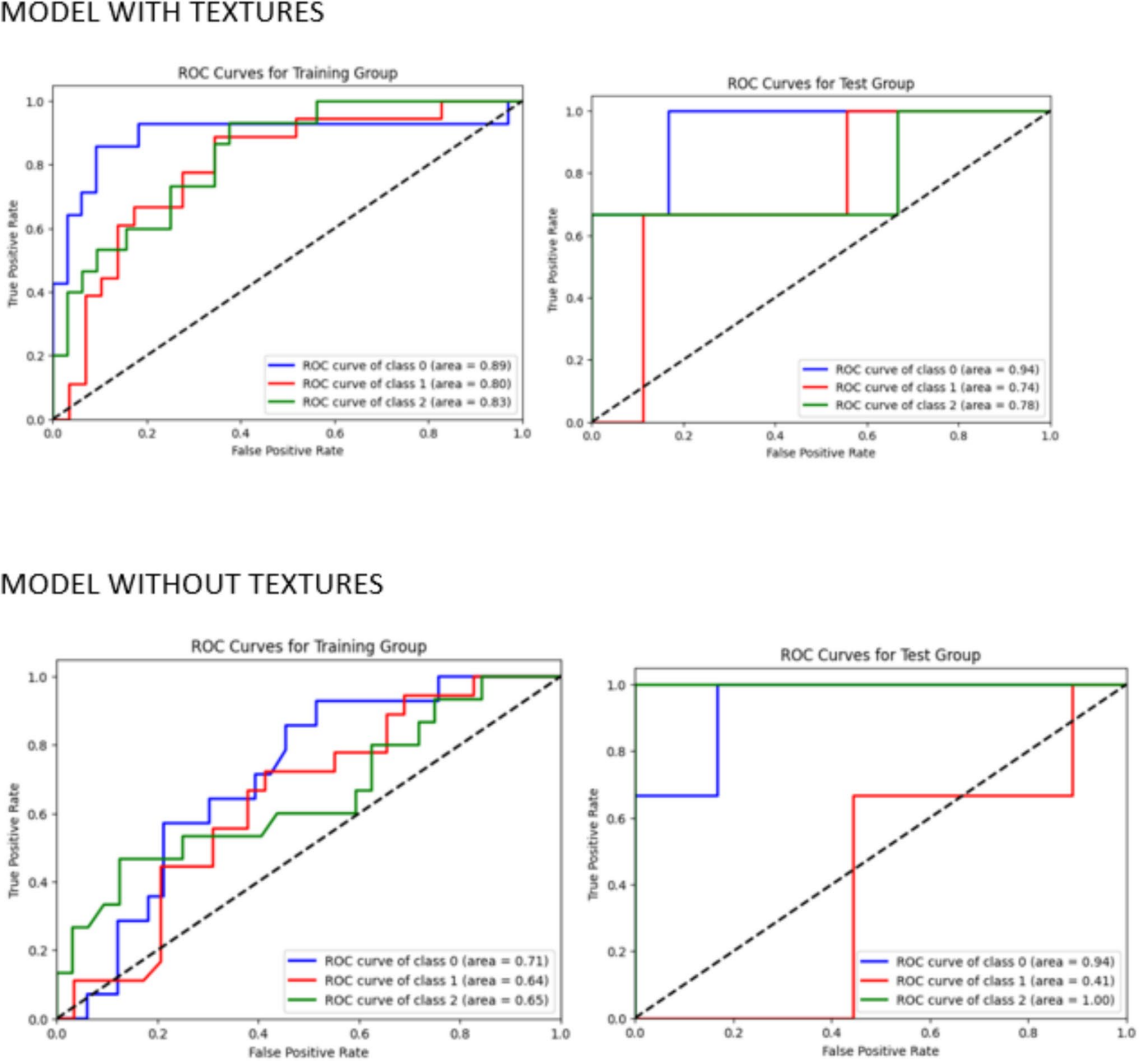
Normal density, osteopenia and osteoporosis classification with and without texture analysis-derived variables. ROC curves for both models’ training and testing groups. Class 0 refers to normal bone density, class 1 to osteopenia and class 2 to osteoporosis

The results of accuracy, precision, recall, F1, and ROC curve for the training group after a tenfold cross-validation analysis and testing on the test group are summarized in Table 2. The ROC curve values for the test group were 0.716, 0.783, 0.833, and 0.916 for the neural network, logistic regression, Naive Bayes, and the ensemble technique, respectively (Table 2). The results obtained for the models classifying the patients with normal bone density, osteopenia and osteoporosis were better for the model including the texture analysis-derived variables compared to the model solely including the HU (accuracies of 0.75 vs. 0.50 and AUC of 0.77 vs. 0.65, respectively). (Table 3, Fig. 2).

**Table 2:** Accuracy, precision, recovery, F1 and ROC curve results for the training and test group of the first machine-learning model

		Neural network	Logistic regression	Naive Bayes	Mixed model neural network and Naive Bayes
Training set	Accuracy	0.645	0.868	0.847	0.881
	Precision	0.816	0.871	0.854	0.884
	Recall	0.70	0.863	0.846	0.880
	F1	0.72	0.868	0.846	0.880
Testing set	Accuracy	0.687	0.812	0.875	0.937
	Precision	0.555	0.800	1.000	1.000
	Recall	0.833	0.666	0.666	0.833
	F1	0.666	0.727	0.800	0.909
	AUC	0.716	0.783	0.833	0.916

**Table 3:** Accuracy, precision, recall, F1 and ROC curve results for the training group and the test group of the second machine-learning model to classify the patients with normal density, osteopenia or osteoporosis

		Neural network (using UH and textures)	Neural network (using only UH)
Training set	Accuracy	0.681	0.489
	Precision	0.693	0.530
	Recall	0.681	0.489
	F1	0.665	0.477
	AUC	0.758	0.612
Testing set	Accuracy	0.750	0.500
	Precision	0.720	0.471
	Recall	0.750	0.500
	F1	0.728	0.464
	AUC	0.769	0.653

## Discussion

In this study, we created a model to predict bone mineral density (BMD) by using four different machine learning methods. These methods analyzed patterns in CT images of the spine's first lumbar vertebra. We found that a combined approach, using a neural network model together with Naive Bayes, was very effective, achieving a high accuracy (an area under the curve or AUC of 0.916) in distinguishing between normal and abnormal BMD. Furthermore, incorporating texture analysis data into the neural network model significantly improved the accuracy in identifying normal bone density, osteopenia, or osteoporosis, more so than using a model based only on the traditional density measurement (HU).

Mean Hounsfield unit (HU) values in patients with osteoporosis were significantly lower than in those with normal bone density, as the measurement of HU values in TC is a method using tissue density of vertebrae trabecular bone mass to represent BMD [16]. Notably, specific HU cutoff points, such as 106 HU with 90% specificity and 61% sensitivity, and 160 HU with 89.9% specificity and 60% sensitivity, demonstrated strong diagnostic potential in distinguishing the presence and absence of osteoporosis compared to DXA. These results align with previous studies by Zou et al. [16] and Pickhardt et al. [13], emphasizing the reliability of HU values in diagnosing osteoporosis. In this last study, the differences in diagnostic yield were analyzed in patients who had undergone CT studies with and without intravenous contrast, without finding significant differences in the AUC between them (0.84 in CT with contrast and 0.83 in those without contrast), with similar accuracy values [13]. These findings are concordants with our study. Although a systematic review published in 2022 [17], which evaluated the effect of contrast media on CT measures of BMD, demonstrated significant differences in BMD values between unenhanced and contrast-enhanced CT scans, we have not found these differences. This could be because, similar to Pickhardt et al. [13], the region of interest we analyzed is located in the center of the vertebral body, far from the main articular and vascular areas, where contrast could have the greatest influence. In addition, our normalization process may have played a role in mitigating potential differences in mean HU values between unenhanced and contrast-enhanced CT scans. Nonetheless, the use of HU values alone showed limitations in differentiating between osteopenia and osteoporosis. The integration of texture analysis variables increased the diagnostic accuracy beyond using the mean HU values alone.

Our results are similar to previous findings in terms of classifying patients with and without osteoporosis. While previous studies explored texture analysis in different anatomical regions, our focus on lumbar spine CT images, typically the site for DXA, adds specificity to osteoporosis prediction [9, 18–20].

Radiomic features such as Kurtosis and gray level non-uniformity, used in previous research [9, 10], were integrated in our predictive model. Kurtosis reflects the intensity distribution peak [11, 21], and gray level non-uniformity measures variability, both significant in assessing bone microstructure alterations due to BMD loss [10, 21]. In our study, despite using a normalization process, we found significant differences in texture analysis when evaluating these bone microstructure alterations, which reinforces its usefulness. Likewise, Mookiah et al. explored the feasibility of opportunistic osteoporosis screening in contrast-enhanced CT scans using texture analysis, and their results demonstrated acceptable reproducibility of texture parameters derived from scans with and without intravenous contrast [22].

Distinct from prior studies on spine pathology, our research focused on patients with bone density changes attributed to primary hyperparathyroidism (PHP) [11, 16, 23]. The increasing availability of CT studies might ease the applicability of texture analysis-derived models, which could minimize costs derived from screening [9, 13].Our study has limitations, including a single-center design with a relatively small sample size. Manual segmentation and image normalization techniques may introduce measurement bias and limit statistical significance, respectively.

Despite these limitations, our study has also several strengths. The consecutive patient recruitment strategy mitigates selection bias. Importantly, our approach, despite limitations, presents a simple and cost-effective screening method for predicting abnormal BMD in patients with PHP, opening avenues for prospective studies to validate its utility in clinical practice.

Furthermore, our exploration of texture analysis, specifically focused on lumbar spine CT images, contributes to the growing body of evidence supporting its applicability in osteoporosis prediction. Radiomic features such as Kurtosis and gray level non-uniformity emerged as crucial components in our predictive models, emphasizing their potential as meaningful indicators of bone microstructure alterations linked to BMD loss. The simplicity and accessibility of our model in clinical practice make it a promising tool for identifying individuals at risk of osteoporosis, guiding further diagnostic decisions, and potentially reducing unnecessary testing costs.

## Conclusion

In conclusion, our study demonstrates the effectiveness of supervised machine learning techniques in accurately classifying normal versus abnormal bone mineral density (BMD) in patients with primary hyperparathyroidism (PHP). Leveraging both clinical features and texture analysis of spinal bone tissue from CT images, our model, particularly the ensemble approach, achieved a notable discriminative power with an AUC of 0.916. This underscores the potential of radiomic features as valuable biomarkers for predicting abnormal BMD.

In addition to the above, our study sets the stage for future prospective investigations to delve deeper into the predictive utility of our approach in patients with primary hyperparathyroidism. Further validation studies with larger cohorts and multicenter designs will enhance the generalizability and robustness of our predictive model. The integration of our model into routine clinical practice has the potential to streamline the diagnostic process for osteoporosis, ultimately improving patient care and optimizing healthcare resource utilization.
